# Parental engagement with complementary feeding information in the United Kingdom: A qualitative evidence synthesis

**DOI:** 10.1111/mcn.13553

**Published:** 2023-08-08

**Authors:** Kelly Spurlock, Toity Deave, Patricia J. Lucas, Sally Dowling

**Affiliations:** ^1^ School of Health and Social Wellbeing University of the West of England Bristol UK; ^2^ Nesta London UK; ^3^ Bristol Medical School University of Bristol Bristol UK

**Keywords:** ‘Complementary feeding’, ‘infant feeding’, ‘infant nutrition’, ‘qualitative’, ‘systematic review’, ‘thematic synthesis’, ‘weaning’

## Abstract

Complementary feeding is the process of introducing solid foods to milk‐fed infants (also known as weaning). Current UK guidance states that complementary feeding should occur around 6 months but not before 4 months. This systematic review explores how parents in the UK, with an infant under 24 months of age, engage with sources of information and advice about complementary feeding. Engaging with sources of information can influence parents' feeding choices and so a better understanding of parents' information behaviours can improve service provisions. Six databases were searched, identifying 15 relevant qualitative studies with the predefined criteria. Data from each study were coded line by line allowing for a synthesis of higher analytical themes. Using thematic synthesis, four main themes were observed: (1) trust and rapport—parents valued information from a trusted source (2), accessibility—information needs were often time sensitive, and parents showed varying levels of understanding, (3) adapting feeding plans—often influenced by practicalities (4), being a good parent—feeding plans were changed to comply with societal ideas of ‘good parenting’. The review concluded that parents receive information and advice about complementary feeding from multiple sources and are highly motivated to seek further information. The scope of this novel review explored the parental experience of finding, receiving and engaging with information sources and how this may or may not have influenced their feeding behaviours. The review has provided a new perspective to add to the growing body of literature that focuses on the experience of feeding an infant.

## Key messages

- This review found that parents held the NHS services in high regard but did not always seek information from healthcare providers due to accessibility issues, preferring the instant nature of online sources.
- Parents were more likely to accept information or advice if a rapport was built and non‐judgemental approach was adopted as it protected the parental identity while providing information about complementary feeding.
- Accessibility of information was important as sources were not always convenient for parents to access or in an understandable format which may reduce adherence to complementary feeding guidelines.

## Graphical abstract

Complementary feeding is the process of introducing solid foods to a milk fed infant's diet. The review synthesised qualitative data that explored how parents in the United Kingdom engage with complementary feeding information and their information needs.

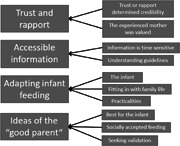

## 1 INTRODUCTION

Nutrition in the first 1000 days has been shown to influence health outcomes in childhood which persist into adulthood (Craigie et al., 2011; Pearce & Langley‐Evans, 2013; Woo Baidal et al., 2016). There is evidence that inappropriate complementary foods and unresponsive feeding practices can alter metabolic processes through epigenetic modification, and they are associated with an increased risk of developing noncommunicable diseases such as heart disease and type 2 diabetes in later life (Edwards et al., 2017; Hetherington, 2016). Eating habits and food preferences during infancy have been shown to persist into adulthood and so complementary feeding is an important window for establishing a healthy and varied diet (Craigie et al., 2011). Additionally, parents who apply pressure to eat can override an infant's innate satiety response which may lead to an unhealthy relationship with food (Breij et al., 2017; Nicklaus & Remy, 2013; Redsell et al., 2016).

Parents typically exercise control over their infant's diet and as main caregivers, they are responsible for decision‐making around their child's diet. They are commonly targeted by health promotion campaigns to improve the quality of their infant's diet (Blake‐Lamb et al., 2016; Redsell et al., 2016). Parents in the United Kingdom (UK) receive information about complementary feeding from multiple sources: the National Health Service (NHS) and healthcare professionals (HCPs), businesses and charities, friends, family or the internet (Garcia et al., 2019). In the UK, the Health Visitor (also known as Specialist Community Public Health Nurse) plays a key role in health promotion for parents with infants and children under 5 years of age and they are often responsible for providing guidance on complementary feeding. Current UK guidance follows the World Health Organisation's global guidelines that solid foods should start at 6 months of age but not before 4 months, and should complement milk feeding rather than replace it (Department of Health, 2003; World Health Organization, 2000). Complementary foods should be suitable for the infant's developmental level and nutritional needs; iron rich foods should be prioritised as requirements at 6 months of age cannot be met with milk alone (Aggett et al., 2002). Foods in the first 2 years of life should focus on unprocessed foods, avoiding foods with high levels of salt and sugar (Department of Health, 2003).

Influences on infant feeding are multiple and interact with each other in a complex manner. Individual studies show that these include practical factors that influence feeding decisions such as cost or lack of time (Harrison et al., 2017; Matvienko‐Sikar et al., 2018; Spyreli et al., 2021); advice and information given by family and friends; guidance from health services and contact with healthcare practitioners (Renfrew et al., 2011); and information provided by the commercial baby food industry (Arden, 2010; Garcia et al., 2019). The emergence of the internet, social media and commercial websites and apps has changed the way parents access information, and may have increased access to information that is not evidence‐based (Cheng et al., 2020; Taki et al., 2015). Understanding how parents access and engage with information about complementary feeding information is important if we want this information to influence their feeding decisions (Gage et al., 2012; Garcia et al., 2019). Systematic reviews, to date, have explored the experience of complementary feeding (Harrison et al., 2017; Matvienko‐Sikar et al., 2018; Spyreli et al., 2021). However, a review that explores parental information seeking behaviours; parents' information needs, and their engagement with information sources has not yet been conducted. The aim of this review was to explore how parents access and engage with information and advice about complementary feeding for their infants under 2 years of age in the United Kingdom.

## 2 METHODS

### 2.1 Review design and identification of studies

A review protocol was developed by [initials removed for review] following the Cochrane guidance for qualitative evidence (Noyes et al., 2022). A protocol was developed and recorded on the Prospective Register of Systematic Reviews (PROSPERO) (REG ID: 168393, February 2022).

Seven bibliographic databases were searched in March of 2022: Medline and PsychINFO, CINAHL, IBSS, Sociological Abstracts and the Web of Science Core collection. The search strategy was adapted for each database, using a search strategy with terms for *complementary feeding* and for *qualitative research* at title and abstract levels and limited to studies published since January 2004. Twenty relevant journals were identified by [initials removed for review] and hand searched for issues published between Jan 2004 and March 2022. The search was limited to include papers published after January 2004 as this marks the point when more than half of UK households (53%) had a home computer with internet access and coincided with the launch of the Netmums.com forum (Netmums.com, 2020; Statista, 2019). Access to the internet changed how parents could access information and support online; the rise of social media has created virtual communities in which parents can share experience and seek advice (Frey et al., 2022; Park et al., 2016).

### 2.2 Selection of studies

The criteria for inclusion were:

1. Qualitative studies that reported the direct experiences of parents experiencing complementary feeding.
2. Parents had healthy full‐term infants under 24 months of age.
3. The study was conducted in the United Kingdom.
4. Published after 2004

The age range of 24 months was selected because complementary feeding occurs within the first 24 months of life (Birch & Doub, 2014; The World Health Organisation WHO, 2004). If a study included parents with infants older than 24 months of age but the data were clearly labelled, the study was included, and the participant data with an infant older than 24 months were not included in the analysis.

Qualitative systematic reviews on complementary feeding were excluded but flagged during the abstract and screening process and were checked for any relevant papers. These were then added to the ‘hand search’ folder. Studies not published in English and lacking ethical approval were also excluded.

EPPI Reviewer web version (UCL institute of Education, 2022) was used for the management of titles and abstracts. Using a sequential checking method, a 5% random sample of studies was screened by [initials removed for review], experienced in conducting systematic reviews, at the beginning of the screening process to ensure the screening processes and inclusion/exclusion criteria were effective. A further 2.5% random sample was screened by [initials removed for review] near the end of the screening phase. All full text papers were reviewed independently by two reviewers, and disagreements resolved by consensus seeking a third opinion when needed. Papers excluded at full text had reasons for exclusion recorded, with all reviewers in agreement (Supporting Information: Data 1).

### 2.3 Quality appraisal and transparency

Eligible studies were appraised by [initials removed for review] using the CASP checklist for qualitative research (CASP‐UK, 2018) (Supporting Information: Data 2). This is used by Cochrane for qualitative synthesis and is suitable for identifying methodological quality (Majid & Vanstone, 2018; Noyes et al., 2018). To enhance transparency in reporting, this review followed the ENTREQ framework (Tong et al., 2012) (Supporting Information: Data 3).

### 2.4 Data extraction and synthesis

Studies were analysed using NVivo 12 (QSR international, 2022) using inductive thematic synthesis, following methods developed by Thomas and Harden (2008). This involved reading repeatedly to begin familiarisation and line‐by‐line coding. Using first order quotes (participant quotes) and second order quotes (author interpretations) studies were coded using line‐by‐line coding. A bank of codes was built enabling the translation of concepts across studies using the existing codes, with new codes being created for new concepts. After coding three studies independently, a meeting was held with [initials removed for review] to discuss coding across the studies. The inductive codes were then developed into descriptive themes and then higher analytical themes. As per Thomas and Harden (2008), whilst remaining rooted in the data with quotes to demonstrate concepts, analytical themes go beyond the original study data to provide an interpretive synthesis to answer the review questions. Analytical themes were discussed with the research team to agree on the overarching conceptual themes.

## 3 RESULTS

**Figure 1:**
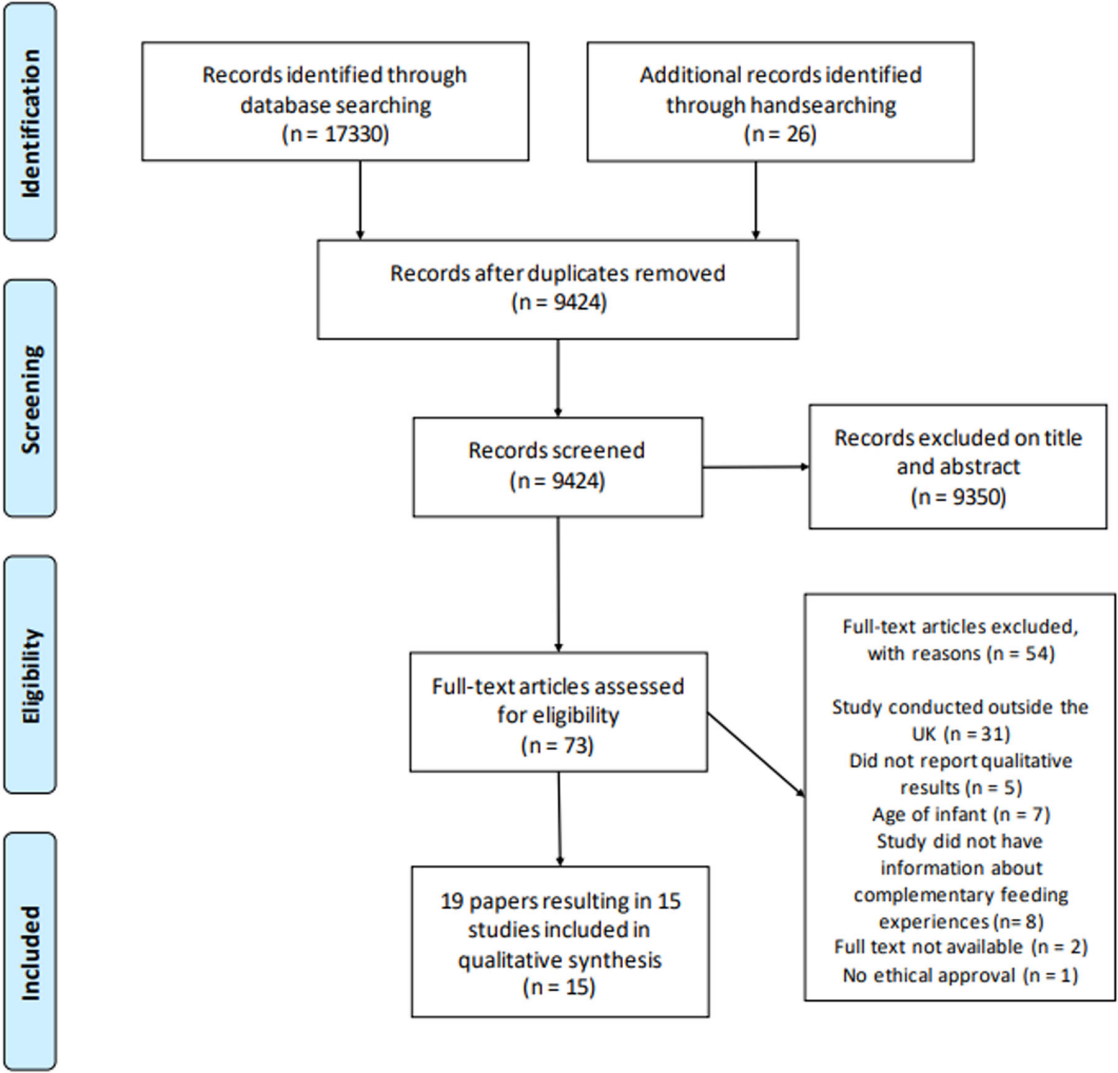
Flow diagram showing the review process adapted from PRISMA (Page et al., 2021).

Seventeen thousand three hundred thirty papers were identified by the search and a further 26 from handsearching. Eighteen papers were eligible for inclusion, four of these papers used data from the same study and were analysed together, resulting in 15 studies for review (see Figure 1).

### 3.1 Characteristics of included studies

The 15 eligible studies reported views from both first‐time and multiparous parents, took place in disadvantaged neighbourhoods and more affluent areas. Two studies focused on migrants and ethnic minorities living in the United Kingdom. Most of the studies included only mothers. Five studies reported fathers in the sample, but the data were limited (study characteristics, Table 1).

**Table 1:** Study characteristics included in the review.

Author and date	Age of infants	Participant	Location	Aim of the study	Methods of data collection and analysis
Andrews et al. (2015)	2.5−6.2 months	15 mothers living in a postcode area designated as deprived by the Scottish index of Multiple Deprivation. Age range 18−44, Primiparous 11, Multiparous 4	Scotland—Tayside	Assessing mothers' knowledge of feeding practices and opinions on current feeding advice after attending 2 NHS weaning workshops.	Semi‐structured 1−1 interviews— Framework analysis
Arden and Abbott (2015)	9−15 months	15 mothers active on parenting sites. Age 29−38 (predominantly breast feeding). 12 primiparous, 3 multiparous	Online—UK—based Internet parenting sites and forums	To investigate mothers' experiences of following baby led weaning.	Semi‐structured interviews via 5 emails embedded with questions—Thematic analysis
Barlow et al. (2010)	3−9 months	8 mothers aged 21‐39 years identified with infants at risk of obesity who had partaken in a health visiting pilot. Identified as BMI in the obese category. Both first time and multiparous.	Leeds	An evaluation of a health visitor service pilot with extra training in obesity prevention	Semistructured 1−1 interviews—Thematic analysis
Brown and Lee (2013)	12−18 months	36 mothers active on parenting sites for BLW. Ages 19−>35, a more affluent and highly educated sample of mothers.	Online‐ UK based	Exploring mothers' experiences of BLW and the decision‐making process to introduce solids	Semi‐structured 1−1 interviews— Content analysis
Carstairs et al. (2017)	5−42 months* mean age of 18.6 months	32 mothers. Mean age 33.7 years (26−44). Equal number of participants from least deprived and most deprived neighbourhoods. Both first time and multiparous.	Scottish Urban/Rural, fishing/nonfishing communities	Exploring the influences on mothers in providing seafood during early years	Q methodology with an accompanying cognitive ‘think aloud’ interview—Descriptive statistics
Caton et al. (2011)	6−18 months	13 mothers with an average age of 28.5 years (20–36). School leaving age (15–21). Average Parity 1.7 (1–4)	Hoyland, Barnsley, South Yorkshire, UK	To explore parental feeding practices relative to official recommendations and to discover how parents introduce vegetables.	Semi‐structured interview**—**Thematic analysis
Garcia et al. (2019)	4−12 months	21 parents, mean maternal age of 30 (range 20−57). Sample from an area of high economic deprivation. 52% of sample living in a deprived household. 90% of the sample had medium−high education. Primiparous and multiparous.	North Lanarkshire, Scotland	explore CF practices of parents and where they found information on CF and what type of information they needed.	Structured 1−1 interviews**—**Descriptive analysis
Hoddinott et al. (2012) and McInnes et al. (2013)	0−6 months	36 mothers and 37 significant others (26 partners, 8 grandmothers, 1 sister and 2 HCPs). Maternal age between ranged from ≤20−40 years. 22 mothers finished fulltime education at ≥19 years. All lived in more deprived areas. Both primiparous and multiparous.	Two health boards who implemented BFI in Scotland around 100 miles apart	To investigate the infant feeding experiences of women and their significant others from pregnancy until 6 months	Serial interviews from prebirth to 6 months—Thematic analysis
Lakhanpaul et al. (2020)	6−23 months	21 mothers, 10 fathers. British‐Bengali nationality living in Tower Hamlets. Primiparous and multiparous.	London Borough of Tower Hamlets	To explore infant feeding practices and their drivers within the British‐Bangladeshi population of East London	Semi‐structured 1−1 interviews with mothers2 focus groups with fathers—Thematic analysis
Lovelace and Rabiee‐Khan (2015)	2−35 months* (Mean—22 months)	12 mothers aged 19−25. 5 single parents. 8 primiparous. 3 were receiving healthy start vouchers.	West Midlands	To explore the food choices made by low‐income families when feeding their pre‐school children; to understand the socioeconomic and environmental influences	Semi‐structured 1−1 interviews—Modified grounded theory approach
McNally et al. (2020)	7–24 month	11 mothers with an average age of 33 years. Nine mothers had an undergraduate degree or higher. All others were from a white UK background. Primiparous and multiparous.	North England	To understand how mothers using different feeding practices (BLW and TW) perceive, understand, and respond to their infants' feeding cues via their reflections on their infants' and their own behaviour during video recordings of TW and BLW interactions.	Semi‐structured interviews—Template analysis
Redsell et al. (2010)	1−11 months Mean—5.51 months	36 mothers, 2 fathers with a mean age of 30.1 years (19−49 years). Participants were mostly white British 29 (76.3%), married 22 (57.9%), employed full‐time 17 (44.7%) and had between 1−5 children.	2 urban sites with high risk of child obesity and 3 areas with high deprivation. 1 area with low deprivation for comparison	Investigate perceptions of infant growth, infant obesity risk and beliefs on weaning practices.	6 focus groups (4−9 participants)—Thematic analysis
Synnott et al. (2007)	3−18 months (mean 8 months)	24 parents aged 25 to ≥35. 22 were married or cohabiting. Primiparous and multiparous.	Glasgow	to gain an insight into parental perceptions of feeding practices in five European countries.	3 focus groups—Content analysis
Tully et al. (2019); Spyreli et al., 2019	3−18 months (mean 8 months)	37 parents who had been identified as with low‐income, low‐educational attainment, one‐parent families, migrants, ethnic minority, <20 years, living in social housing/direct provision, unemployed or with little/no support were targeted.	Northern Ireland, Belfast and 2 areas outside, disadvantaged communities	Explore the knowledge, attitudes and practices of parents living in disadvantaged areas of Northern Ireland and the key sources of information.	8 focus groups (2−7 participants)—Thematic analysis
Zhang et al. (2020); Zhang and Benton 2019	6−24 months	14 mothers who migrated from mainland China with a mean age of 29 years. Mostly housewives living in the UK for an average of 10 years. First time and multiparous.	London, Chinese communities	Explore the effects of culture on infant care for mothers born in China living in the UK	1−1 semistructed Interviews**—**Thematic analysis

### 3.2 Sources of information

Parents across the studies received complementary feeding information and advice from multiple sources, as shown in Table 2.

**Table 2:** Sources of information received by parents.

Author and date	Sources of information
Andrews et al. (2015)	Commercial baby food literature, Weaning workshop (NHS provided), peer support, internet (general), internet (forums), family & friends, perceived infant cues
Arden and Abbott (2015)	Health visitor, books, perceived infant cues
Barlow et al. (2010)	Health visitor, weighing of the infant (indicator of growth and feeding success),
Brown and Lee (2013)	Perceived infant cues,
Carstairs et al. (2017)	Healthcare professional (not‐specified), Health visitor, NHS provided literature, the media (not‐specified)
Caton et al. (2011)	Health visitor, books, family & friends, parenting magazines,
Garcia et al. (2019)	Healthcare professional (not‐specified), NHS provided literature, NHS website, Health visitor, internet (general), internet (forums), Weaning workshop (NHS provided), internet (youtube), Facebook parenting groups, private paid weaning workshops,
Hoddinott et al. (2012); McInnes et al. (2013)	Healthcare professional (not‐specified), Health visitor, friends & family, family abroad in home country, perceived infant cues, perceived infant cues
Lakhanpaul et al. (2020)	Commercial baby food literature, Healthcare professional (not‐specified), Health visitor, fathers preferred to get advice from the doctor (General Practitioner), Children's centres, internet (general), community groups, Weaning workshop (NHS provided), peer support, perceived infant cues
Lovelace and Rabiee‐Khan (2015)	Commercial baby food literature, Health visitor, doctor (General Practitioner), friends & family, books, perceived infant cues
McNally et al. (2020)	Health visitor, perceived infant cues
Redsell et al. (2010)	Healthcare professional (not‐specified), Health visitor, perceived infant cues,
Synnott et al. (2007)	internet (general), family & friends, Healthcare professional (not‐specified), Health visitor, perceived infant cues
Tully et al. (2019); Spyreli et al. (2019)	Commercial baby food literature, Healthcare professional (not‐specified), Health visitor, weighing of the infant (indicator of growth and feeding sucess), internet (forums), internet (general), social media, books, perceived infant cues
Zhang et al. (2020); Zhang and Benton (2019)	Health care professional (not‐specified), midwives, doctor (General Practitioner), Family & friends, internet (mothers group), Health visitor, children's centre, NHS website, peer support groups, family abroad in home country, social media, books

**Figure 2:**
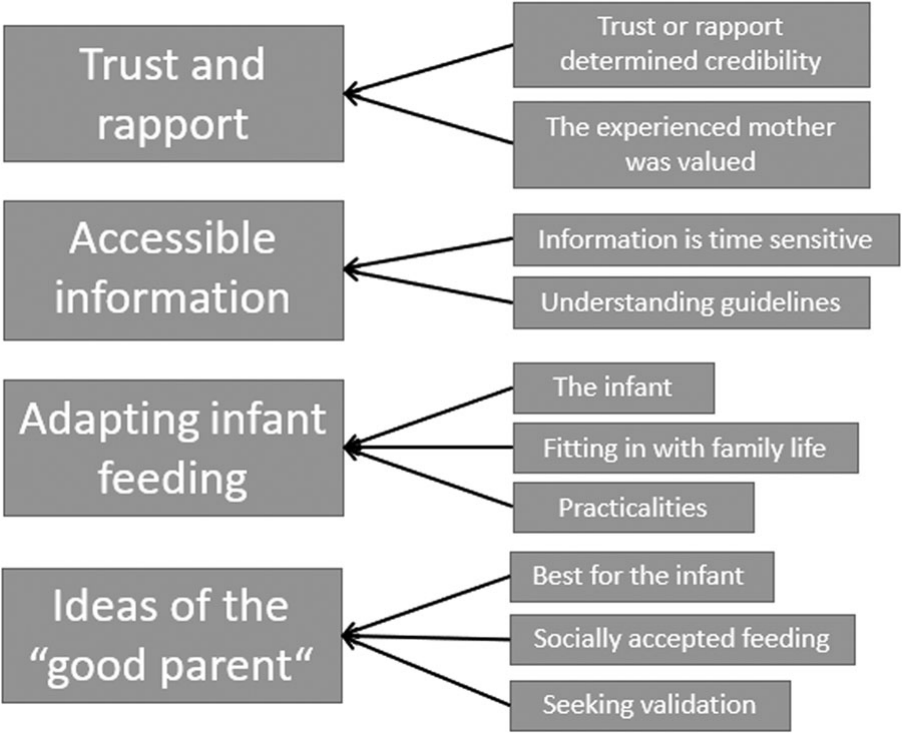
Diagram showing the main themes and subthemes.

Analysis of the 15 studies resulted in four themes: trust and rapport, accessibility of information, complying to ideas of good parenting and adapting feeding plans (see Figure 2). Quotes to support each theme can be seen in Table 3.

**Table 3:** Quotes illustrating analytical themes and subthemes.

Theme	Quote	Source
Trust and rapport**—**Trust influenced credibility of sources	‘Quite nice for it to be someone that perhaps you've got a bit of a relationship with and I don't know I mean I saw a lot of the health visitor when [baby's name] was very little and would have welcomed her and did welcome any advice that she had for me and she I felt that she actually knew [baby's name] quite well’.	(Redsell et al. [2010], p.7)
Trust and rapport**—**Trust influenced credibility of sources	‘It was also apparent that, if respondents felt the workshop staff had discredited or dismissed their opinion on one issue, then it was difficult to accept any advice from them’.	(Andrews et al. [2015], p.565).
Trust and rapport**—**Trust influenced credibility of sources	‘That's when the stage 1(baby food) jars started’.	(Lovelace & Rabiee‐Khan [2015], p.874).
Trust and rapport**—**Trust influenced credibility of sources	‘Because I thought it was the right thing to do because it was Heinz (said with added emphasis) and it was like, it was £2 a bottle…’	(Lovelace & Rabiee‐Khan [2015], p.874).
Trust and rapport**—**The experienced mother	‘Yeah a lot of health visitors don't have children. And it's okay to turn around and say it from a leaflet, you know … but they're not the ones pulling their hair out at two o'clock in the morning, when they haven't had sleep for six months’.	(Tully et al. [2019], p.6).
Trust and rapport**—**The experienced mother	‘I did it that way because my mum advised…’	(Caton et al. [2011], p.882).
Accessibility**—**Information is time sensitive	‘But I think the one I went back to was the one I really learned most at, because I knew what I was trying to achieve at that point’	(Andrews et al. [2015], p.564).
Accessibility**—**Information is time sensitive	‘It's easier to access as well, its quicker to access information online than it is to contact a health professional about it and wait until they get back to you’	(Garcia et al. [2019], p.10).
Accessibility**—**Understanding guidelines	‘… with things like chocolate… we try to avoid that because obviously it's gonna rot his teeth… we usually give him a couple of rusks or crisps… things like that’	(Lovelace & Rabiee‐Khan [2015], p.874).
Accessibility**—**Understanding guidelines	‘Sometimes I read too that what you ate in pregnancy can affect what your baby eats but I don't know if that's the thing or not’.	(Spyreli et al. [2019], p.8).
Adapting feeding plans—Infant cues	‘She took a piece of cucumber out of my hand and shoved it in her mouth so I took that as a sign she was ready’	(Brown & Lee [2013], p.236).
Adapting feeding plans—Infant cues	‘I started to wean her because she started to wake up at night’.	(Redsell et al. [2010], p.5).
Adapting feeding plans—Fitting into family life	‘I feed her first cause then she's kind of quiet and content and try and finish the rest (of the children)’.	(Spyreli et al. [2019], p.13).
Adapting feeding plans—Fitting into family life	‘… always sit up to the table and eat with your baby don't get worked up some days he will eat more than others just like us so don't worry and enjoy it…’	(Arden & Abbott [2015], p.836)
Adapting feeding plans—Fitting into family life	‘She has eaten with us from around 7 months. We changed her routine so we could all eat together in the evening when her dad gets home’.	(Brown & Lee [2013], p.236).
Being a good parent**—**Best for the infant	‘I think it's good to ha'e a recommendation, because you don't want to hurt the wee one either, you know, their wee stomachs and that’.	(Andrews et al. [2015], p.561).
Being a good parent**—**Best for the infant	‘100% salmon, 100% haddock, 100% cod, took the breadcrumbs off to reduce salt intake… I promised myself that I would ensure that xxx had a varied diet with as much fruit and vegetables as I could’.	(Caton et al., [2011], p.821).
Being a good parent**—**Best for the infant	‘Cos it is organic and, you know, somehow’ that's much better, you know?’	(Tully et al. [2019], p.8).
Being a good parent**—**Best for the infant	‘I don't think there's anything worse than a fussy child, when it's you know I don't like that I don't want it I don't want it, it can get you really stressed’.	(Spyreli et al. [2019], p.9).
Being a good parent**—**Socially accepted feeding	‘I think sometimes it's because they want them to sleep through—I think it is laziness on the mum's part’.	(Caton et al. [2011], p.823).
Being a good parent**—**Socially accepted feeding	‘I've watched babies scream at the dinner table whilst being forced fed some mush, but we've always had fun at dinner time’	(Arden & Abbott [2015], p.837).

### 3.3 Trust and rapport

The theme of trust and rapport was important in respect of acceptance of information and featured consistently across papers.

#### 3.3.1 Trust influenced credibility of sources

Parents followed advice and information that came from sources they trusted and trust could be built in a number of different ways. In several studies, the importance of the relationship with the individual giving advice was stressed. This was seen with HCPs who were trusted as credible sources of information when there was a rapport with the family and they were viewed as nonjudgmental (Andrews et al., 2015; Barlow et al., 2010; Hoddinott et al., 2012; McInnes et al., 2013; Redsell et al., 2010; Zhang et al., 2020). If there was a lack of rapport or distrust in the source of information, parents reported hiding their feeding behaviours and stopped seeking advice from this source. This was also the case if the information provider had dismissed the parents' ideas or opinions. Parents reported feeling overwhelmed when they did not know how to assess credibility or which advice to trust (Andrews et al., 2015; Hoddinott et al., 2012; Tully et al., 2019). Commercial sources of information, such as books by authors who were regarded as experts, were used by parents because they trusted the advice given and their advice was felt to replace other guidelines (Arden & Abbott, 2015; Tully et al., 2019). Age‐specific information provided on baby food packaging was interpreted as guidance so could also influence a parent's feeding behaviours (Lakhanpaul et al., 2020; Lovelace & Rabiee‐Khan, 2015). When questioned why a mother introduced food at 4 months she responded:

‘That's when the stage 1(baby food) jars started’.—(Lovelace & Rabiee‐Khan, 2015, p.874).

A parent's trust and loyalty to a food brand could influence them to purchase products without questioning the nutritional quality.

#### 3.3.2 The experienced mother

Mothers trusted and valued the advice of experienced mothers very highly, sometimes more than advice from a HCP if the HCP did not have children of their own. Advice from maternal grandmothers was often seen as superior to information provided by HCPs, and mothers would consult their own mothers about complementary feeding information:

‘I did it that way because my mum advised…’—(Caton et al., 2011, p.882).

Mothers would seek information from other mothers they knew, such as sisters or friends, or actively seek mothers online in forums or in magazines with parenting tips (Andrews et al., 2015; Caton et al., 2011; Garcia et al., 2019; Hoddinott et al., 2012; Lakhanpaul et al., 2020; Lovelace & Rabiee‐Khan, 2015; Tully et al., 2019; Zhang et al., 2020). Mothers often sought the advice of experienced mothers to meet their information needs on specific topics such as allergies, choking or practical information about portion sizes or recipes when they felt information provided by formal sources was lacking (Andrews et al., 2015; Caton et al., 2011; Garcia et al., 2019; Zhang et al., 2020).

### 3.4 Accessibility—timing and clarity of information

The second theme was the accessibility of information. This encompassed the availability of information and if the information was provided in a format that was easy to understand for parents.

#### 3.4.1 Information is time sensitive

Parents wanted complementary feeding information at the right time (Andrews et al., 2015; Barlow et al., 2010; Garcia et al., 2019; Tully et al., 2019). Parents' information needs for complementary feeding information changed over time as parents progressed through the different stages of feeding. A crucial point to provide information was before the parent had introduced complementary foods; if given then it could prevent the early introduction of solids or poor feeding practices (Andrews et al., 2015; Barlow et al., 2010; Tully et al., 2019). Parents also appreciated ongoing support when challenges arose when they began complementary feeding and the timing of ‘weaning workshops’ was important. Parents also required information quickly and this is where social media and the internet were valued because parents did not want to wait for a response from a HCP. Parents requested online videos from NHS sources as they found the video format more accessible than attending a workshop. They reported that it was difficult to assess credibility of feeding information on platforms such as YouTube (Garcia et al., 2019).

#### 3.4.2 Understanding guidelines

The second subtheme within the theme of accessibility was the clarity of information and if the information was presented in an understandable format. Parents knew the current guidelines but not all parents understood how to follow these guidelines or the rationale behind them. One key example of parents showing a misunderstanding of guidelines was in the study by Lovelace and Rabiee‐Khan (2015) who highlighted that parents were providing unsuitable foods high in sugar, salt and fats under the belief they were providing a healthy balanced diet:

‘… with things like chocolate… we try to avoid that because obviously it's gonna rot his teeth… we usually give him a couple of rusks or crisps… things like that’**—**(Lovelace & Rabiee‐Khan, 2015, p.874).

Parents showed varying knowledge of the rationale behind the guidelines, with some parents showing uncertainty which, in turn, could impact whether they chose to follow the recommendations.

### 3.5 Adapting feeding plans

The third theme, adapting feeding plans as parents navigated the challenges of feeding an infant with everyday pressures, was a common theme across studies. Feeding challenges could cause a parent to seek new information or to not follow previously accepted information.

#### 3.5.1 Infant cues

The infant themselves influenced parents' feeding plans and infant behaviours were used as a source of information which could influence the introduction of solids, either through the infant grabbing food or by the parent interpreting baby behaviour as cues and signs of readiness. An infant's night‐time sleeping habits were often used as a cue for the introduction of solids (Caton et al., 2011; Hoddinott et al., 2012; McInnes et al., 2013):

‘I started to wean her because she started to wake up at night’.—(Redsell et al., 2010, p.5).

Infant illness could be used as a reason why not much food was consumed as could the infant's state (tiredness) or general temperament.

#### 3.5.2 Fitting into family life

Families had to navigate feeding an infant and adapting feeding behaviours to fit in with existing routines. Parents would choose a feeding method that reduced stress at mealtimes. For example, to minimise distractions and save time, some parents found it easier to feed the baby alone in a highchair before the family meal. Other families preferred to feed the infant at the table with the family as this was seen as easier and encouraged the social aspects of eating. These choices were often influenced by how many children were already in the household and convenience. In all the studies, choices about complementary feeding were typically made by the mother, but fathers could influence complementary feeding practices direct through feeding the child, or indirectly (Arden & Abbott, 2015; Brown & Lee, 2013; Carstairs et al., 2017; Hoddinott et al., 2012; McInnes et al., 2013), for example, waiting for his return from work, thus dictating mealtimes. Parents adapted their feeding practices to reduce stress at mealtimes and encourage infants to eat more: using screens at mealtimes or using distraction techniques, such as singing or allowing toys at the table (Lakhanpaul et al., 2020; McNally et al., 2020; Spyreli et al., 2019).

#### 3.5.3 Practicalities of following guidelines

Practical barriers to following advice and often beyond their control and included factors such as cost (Andrews et al., 2015; Brown & Lee, 2013; Carstairs et al., 2017; Lovelace & Rabiee‐Khan, 2015; Redsell et al., 2010), cooking ability (Lovelace & Rabiee‐Khan, 2015; Redsell et al., 2010; Synnott et al., 2007), mother returning to work (Hoddinott et al., 2012; McNally et al., 2020; Tully et al., 2019), practical social support (Tully et al., 2019) and breastfeeding problems (McInnes et al., 2013). Availability of food, such as locality of food shops or foods that were culturally accepted within the household, could influence how an infant was fed. This might be negative as families could be influenced by fast‐food outlets in the local area (Lakhanpaul et al., 2020). It could also have a positive effect on an infant's diet as parents would see healthy alternatives being offered at day‐care and playgroups and, if their infant ate them, they would serve them at home. In one example, a mother disclosed that the infant did eat vegetables and fruit but only at the grandmother's home as they did not offer them at home (Lovelace & Rabiee‐Khan, 2015).

### 3.6 Being a good parent

The final theme was complying with the idea of being a good parent. Parents often linked feeding decisions with the construct of the ideal parent and being able to care for and support the health of their infant.

#### 3.6.1 Best for the infant

All studies found that parents felt compelled to do the best for their children, which motivated them to actively seek information about complementary feeding to meet their information needs. Many parents expressed an appreciation for the guidelines as they were motivated to do the best for their infant and prevent harm. Parents were motivated to provide a good and balanced diet for their infants and this influenced their feeding behaviours. Parents often described a healthy diet as one full of fruits and vegetables and a restricted amount of high fat and sugar foods, demonstrating retention of health information. There was little mention of other nutrients such as carbohydrates or protein. There was also an emphasis on organic foods being better but there was no elaboration about why this was. The rationale behind following a good diet was not always fully understood (Lovelace & Rabiee‐Khan, 2015; Synnott et al., 2007). Some parents showed an awareness of eating habits persisting into later life (Redsell et al., 2010; Spyreli et al., 2019). A common topic that came up was fussy eating and how to prevent this, it was seen as negative and something that could be prevented through a varied diet in infancy.

#### 3.6.2 Socially accepted feeding

Parents felt they were required to feed in a socially accepted way and would adapt their feeding methods in public, due to fear of judgement and embarrassment, or hide their feeding plans to those who might disapprove. This was common with mothers who were following Baby Led Weaning (BLW) as they described the method as ‘alternative’ or too messy for outside the home (Arden & Abbott, 2015; Brown & Lee, 2013). There was an idea that good parenting and feeding were linked, and mothers reported fearing shame and guilt for their feeding decisions (Arden & Abbott, 2015; Brown & Lee, 2013; Caton et al., 2011; Hoddinott et al., 2012; Lakhanpaul et al., 2020; McInnes et al., 2013; Tully et al., 2019) as shown by this quote linking early introduction of solids to laziness:

‘I think sometimes it's because they want them to sleep through—I think it is laziness on the mum's part’.—(Caton et al., 2011, p.823).

Some mothers linked traditional complementary feeding to force‐feeding and that BLW was superior. The dichotomous presentation of feeding with BLW described as ‘right’ and ‘natural’ and spoon feeding as negative or harmful suggests there is a moral dimension to complementary feeding (Arden & Abbott, 2015; Brown & Lee 2013).

#### 3.6.3 Seeking validation

All studies found that parents sought validation for their feeding choices to protect their parental identity and confirmed that they were doing the best for their infant. This was apparent when parents went against infant feeding guidelines. Parents reported that guidelines were too simplistic and too general to be followed. A common justification parents used was that every baby was different, describing guidelines as a ‘one size fits all’ approach which lacked practical and tailored information. Parents described searching for information that validated how they were feeding their infants and they could ignore information from sources that opposed their beliefs (Andrews et al., 2015; Garcia et al., 2019; Lakhanpaul et al., 2020; McInnes et al., 2013; Tully et al., 2019; Zhang et al., 2020).

Parents rationalised their feeding decisions by referring to their baby's behaviours or needs. Grabbing food could provoke the early introduction of solids, perceived hunger could influence portion size. Mothers who were following BLW reported trusting their infant to regulate their appetite. Mothers shared concerns that their infant was not getting enough nutrients, often using the phrase, ‘food under one is just for fun’, as a justification for their infant consuming small amounts (Arden & Abbott, 2015). Some mothers believed that milk, especially breastmilk, was sufficient until an infant reached their first birthday and that solid foods were not important. This contradicts current guidance (Arden & Abbott, 2015; Brown & Lee, 2013; Lakhanpaul et al., 2020; Tully et al., 2019).

## 4 DISCUSSION

This is the first evidence synthesis to explore how parents engaged with sources of information about complementary feeding. This review adds to a body of work that explores the experience of feeding an infant (Harrison et al., 2017; Matvienko‐Sikar et al., 2018; Spyreli et al., 2021). Four themes were identified: trust and rapport, accessibility of information, adapting feeding and ideas of good parenting. This review highlighted similar areas to previous studies: initiating complementary feeding can be a stressful period for parents who required information to match their infants' stage of feeding (Harrison et al., 2017; Nielsen et al., 2013). Seeking information and advice could reduce these stresses and provide confidence provided that complementary feeding information and advice was provided in a non‐judgemental timely manner. Parents reported that formal sources of information, such as ‘weaning classes’, were not always signposted, suggesting that health services could promote available support more effectively. As highlighted in previous studies, parents can misinterpret normal infant behaviour, such as showing an interest in food or night waking, as signs of developmental readiness for solids. This might encourage the early introduction of complementary foods and overfeeding (Arden, 2010; Begley et al., 2019; Schwartz et al., 2013; Swanepoel et al., 2020; Walsh et al., 2015). Future health promotion campaigns or interventions to improve infant feeding could focus on helping parents to recognise these normal infant behaviours that should not cause concern (Daniels et al., 2009; 2015).

The review found that parents can describe feeding guidelines but not always understand or know what constitutes a healthy diet and they did not always follow recommendations. Reasons included lack of trust of healthcare practitioners and practical aspects or influences from friends and family. Parents, particularly those on lower incomes, were influenced by baby food companies due to a trust of the brand and could interpret product suggested age ranges displayed on packaging as infant feeding advice (Andrews et al., 2015; Lovelace & Rabiee‐Khan, 2015). This is worrying because commercially available baby foods are often highly‐processed, high in sugars and salt and frequently more expensive than similar products not marketed for infants (Garcia et al., 2016; Tedstone et al., 2019). Baby food manufacturers influence mothers' feeding decisions because mothers place trust in brands for feeding advice (Palmer, 2011; Tully et al., 2019). A qualitative study of 24 UK mothers reported a belief that mass‐produced baby foods were safer and more nutritious than homemade foods, which is in direct opposition to current UK guidance (Maslin et al., 2015). The influence baby food companies may have on parents should be considered when thinking about how to best advise parents on complementary feeding. The review found that parents were influenced by commercial sources, such as books and industry, and chose to engage with these sources of information. This finding has also been found in more recent papers (Arden & Abbott, 2015; Garcia et al., 2019; Tully et al., 2019). Previous reviews have suggested that parents view family and friends as the most trusted sources and after this, health care practitioners and finally food companies. This suggests that food companies and industry have increased their engagement with parents (Matvienko‐Sikar et al., 2018). This change may have implications as parents chose to follow advice from industry when advice from healthcare practitioners was not seen as a convenient option.

A novel finding in this review was that parents described being highly motivated to seek information and would look for information that suited their preconceived beliefs of what a good parent looked like (Andrews et al., 2015; Garcia et al., 2019; Lakhanpaul et al., 2020; McInnes et al., 2013; Tully et al., 2019; Zhang et al., 2020). This review also highlighted parents' need for practical information that could help them to navigate complementary feeding and for support to be effective it needed to be given at the right time. One way of doing this has been studied in a randomised controlled trial (Daniels et al., 2014; 2015). This aimed to teach parents how to anticipate feeding challenges that might arise and how to overcome them and was successful in increasing parental self‐efficacy and increasing variety of infants' diets. Trust, rapport and experience were key components to parents accepting new information and these align with previous findings in which mothers valued a non‐judgmental approach with continuity of care (Morton, 2020). Complementary feeding is a transition and has different stages which present new challenges for parents (Harrison et al., 2017). This review was the first to highlight the importance of parents receiving information at the right time and that if crucial windows of opportunity were missed parents would seek information from other sources, that may not be evidenced‐based. A novel finding from this review was that parents requested information in video format (e.g., YouTube) that could be accessed at a time convenient to them. Another novel finding was that parents relied on using social media when healthcare practitioners were not accessible, which was not reflected in previous studies. It is possible that the COVID‐19 pandemic and lack of access to healthcare services changed how parents find and use information, with more turning to online resources (Chee et al., 2023). The effect of social media on parents' complementary feeding choices requires further investigation and should be a consideration for future health promotion schemes due to the risks of misinformation (Chee et al., 2023; O'keeffe et al., 2020).

Most included studies focused on the maternal experience, viewing them as the main decision‐makers for their babies (Harrison et al., 2017; Matvienko‐Sikar et al., 2018; Spyreli et al., 2021). It was clear that fathers included in these studies shared the sense of obligation to provide their infant with a good diet, but data were not available to fully explore whether fathers seek information in the same way mothers do, or if they felt the same psychosocial influences. Further work is needed to explore how fathers look for and use complementary feeding information and if it differs to the mother's experience.

### 4.1 Strengths and limitations

The strengths of this review lie in the methodology and rigour with which it was conducted. The comprehensive searching was extensive with screening conducted independently by three reviewers, each paper being reviewed twice. The results show clear links to the original studies through use of quotes as examples for each theme. Including only UK studies reflects the experience of parenting within British culture, with access to a universal healthcare service, although this does reduce generalisability. A limitation was the lack of data available about engaging with sources of information as this was not the main focus for the majority of the original papers. A further limitation was that fathers were not included in most of the original studies, and so the father's role in infant feeding decisions is underrepresented, (Harris et al., 2020; Khandpur et al., 2014; Matvienko‐Sikar et al., 2018) but they have been found to have both indirect and direct influences on childcare (Anderson et al., 2010; Hounsome & Dowling, 2018).

## 5 CONCLUSION

This novel review synthesised the findings of 15 papers to provide an insight into how parents engage with sources of information. Parents in this review held the NHS in high regard but did not always seek advice from healthcare professionals due to accessibility difficulties and preferring an online approach when seeking advice; this could lead them to non‐evidenced based sources of information. Parents received information about complementary feeding from various sources; by understanding how parents engage with sources of information and advice these can be provided in a more accessible way. The findings of this review will inform how advice about complementary feeding can be provided in the future and has relevance to both healthcare providers and stakeholders when designing services. The findings of this review suggest that health services that provide information about complementary feeding need to adapt to provide practical skills to enable parents to follow guidelines.

## AUTHOR CONTRIBUTIONS

Kelly Spurlock, Patricia J. Lucas, and Toity Deave performed the research. Kelly Spurlock, Patricia J. Lucas, Toity Deave and Sally Dowling designed the research questions, search strategy and methods. Kelly Spurlock performed the searches, Kelly Spurlock, Patricia J. Lucas and Toity Deave screened the papers, Kelly Spurlock completed the critical appraisal and Kelly Spurlock performed the analysis supervised by Toity Deave and Patricia J. Lucas. Kelly Spurlock wrote the paper and Toity Deave and Sally Dowling edited it. All authors have read and approved the final manuscript.

## CONFLICT OF INTEREST STATEMENT

The authors declare no conflict of interest.

## ETHICS STATEMENT

As there were no human participants, ethical approval was nonapplicable.

## Supporting information

Supporting information.

Supporting information.

Supporting information.

## Data Availability

Data sharing is not applicable to this article as no new data were created or analysed in this study.
