# Supplementary material for: Parental engagement with complementary feeding information in the United Kingdom: A qualitative evidence synthesis
Source: Matern Child Nutr. 2023 Aug 8;19(4):e13553. doi: 10.1111/mcn.13553 (PMC10483955; doi:10.1111/mcn.13553)
Supplement: Supplementary file 1 — Supporting information. [file MCN-19-e13553-s001.docx]

Supplementary data 1. Table of excluded studies

| Study name | First author (date) | Reason for Exclusion |
| --- | --- | --- |
| Conflicting influences on UK mothers' decisions to introduce solid foods to their infants | Arden (2010) | Age – Cannot distinguish participants with infants under 24 months |
| Relationships between paternal attitudes, paternal involvement, and infant-feeding outcomes: Mixed-methods findings from a global on-line survey of English-speaking fathers | Atkinson et al. 2021 | Topic – Did not focus on complementary feeding |
| Postnatal care: exploring the views of first-time mothers | Bailey (2010) | Topic – Did not focus on complementary feeding |
| "I would rather be told than not know" - A qualitative study exploring parental views on identifying the future risk of childhood overweight and obesity during infancy | Bentley et al. (2017) | Topic – Focused on identifying infant obesity not information sources |
| Peanut exposure during pregnancy, breastfeeding and complementary feeding: perceptions of practices in four countries | Boulay et al. (2015) | Age – Cannot distinguish participants with infants under 24 months |
| Mothers' experiences of feeding situations - an interview study | Bramhagen et al. (2006) | Country – Study conducted in Sweden |
| Key theoretical frameworks for intervention: understanding and promoting behavior change in parent-infant feeding choices in a low-income population | Brophy-Herb et al. (2009) | Country – Study conducted in USA |
| Healthcare professionals' and mothers' knowledge of, attitudes to and experiences with, Baby-Led Weaning: a content analysis study | Cameron et al. (2012) | Country – Study conducted in New Zealand |
| Factors influencing mothers' decisions on whether to provide seafood during early years' feeding: A qualitative study | Carstairs et al. (2017) | Age - Cannot distinguish participants with infants under 24 months |
| What parents want to know in the first postnatal year: A Delphi consensus study | Cashin (2021) | Country – Study conducted in Australia |
| You likes your way, we got our own way': Gypsies and Travellers' views on infant feeding and health professional support | Condon (2015) | Age – Cannot distinguish participants with infants under 24 months |
| Parents' experiences of complementary feeding among a United Kingdom culturally diverse and deprived community | Cook (2021) | Age – Cannot distinguish participants with infants under 24 months |
| Introducing Complementary Foods to Infants: Does Age Really Matter? A Look at Feeding Practices in Two European Communities: British and Italian | Costantini (2018) | Methods - Qualitative data not presented only quantitative |
| Digital distraction or accessible aid? Parental media use during feedings and parent-infant attachment, dysfunction, and relationship quality | Coyne (2022) | Country – Study conducted in the USA |
| Study name | First author (date) | Reason for Exclusion |
| Parent's decisions about starting and ending a meal for their child during infancy, weaning and preschool: A cross-cultural qualitative study | Dobrescu (2022) | Not available – conference abstract |
| Infant and maternal predictors of early life feeding decisions. The timing of solid food introduction | Doub (2015) | Country – Study conducted in the USA |
| Choking, allergic reactions, and pickiness: A qualitative study of maternal perceived threats and risk avoidance strategies during complementary feeding | Dorsey-Graf (2022) | Country – Study conducted in the USA |
| Complementary feeding: A practice between two knowledges | Forero (2018) | Country – Study conducted in Bogota, South America |
| Sources of feeding advice in the first year of life: who do parents value? | Gildea (2009) | Methods - Qualitative data not presented only quantitative |
| People try and police your behaviour': the impact of surveillance on mothers and grandmothers' perceptions and experiences of infant feeding | Grant (2018) | Topic – Study did not mention complementary feeding |
| Understanding health behaviour in pregnancy and infant feeding intentions in low-income women from the UK through qualitative visual methods and application to the COM-B (Capability, Opportunity, Motivation-Behaviour) model | Grant (2019) | Topic – Study did not mention complementary feeding |
| RAISE (Raising Infants to Be Smart Eaters) Pilot Study | Hale (2022) | Country – Study conducted in Canada |
| What's the fuss about? Parent presentations of fussy eating to a parenting support helpline | Harris (2018) | Country – Study conducted in Australia |
| Navigating motherhood and maternal transitional infant feeding: Learnings for health professionals | Harrison (2018) | Country – Study conducted in Australia |
| A qualitative study exploring parental accounts of feeding pre-school children in two low-income populations in the UK | Hayter (2015) | Topic – Study did not mention information seeking about complementary feeding |
| Barriers to compliance with infant-feeding recommendations among low-income women | Heinig (2006) | Country – Study conducted in the USA |
| Feeding Infants and Toddlers: A Qualitative Study to Determine Parental Education Needs | Heller (2019) | not available |
| Development and Qualitative Pretesting of Child Feeding and Obesity Prevention Messages for Parents of Infants and Toddlers | Heller (2021) | Country – study conducted in USA |
| Study name | First author (date) | Reason for Exclusion |
| Maternal decisions about the initiation and termination of infant feeding | Hodges (2008) | Country – study conducted in USA |
| The associations of psychosocial factors and infant feeding beliefs and practices of young, first time, low income mothers | Karp (2010) | Country – study conducted in USA |
| Knowledge on the Complementary Feeding of Infants Older than Six Months among Mothers Following Vegetarian and Traditional Diets | Kostecka (2021) | Country – study conducted in Poland |
| Cultural considerations in postnatal dietary and infant feeding practices among Chinese mothers in London | Leung (2017) | Ethical - Does not state that ethical approval was sought or given |
| A qualitative insight into informal childcare and childhood obesity in children aged 0-5 years in the UK | Lidgate (2018) | Age – Cannot distinguish participants with infants under 24 months |
| [Eating habits in the first year of life: social representations of young mothers] | Lima (2014) | Country – Study in Brazil, article in Portuguese |
| Factors Influencing Parental Engagement in an Early Childhood Obesity Prevention Program Implemented at Scale: The Infant Program | Love (2018) | Country – Study in Australia |
| How parents process child health and nutrition information: A grounded theory model | Lovell (2016) | Country – Study in USA |
| In-depth mental health evaluation of a community sample of nonreferred infants with feeding difficulties | Maldonado-Duran (2008) | Country – Study in USA |
| Feeding Habits in the Cultural Domains of Child Care: Elements for Health Promotion | Martinez-Lopez (2021) | Country – Study in Mexico |
| Sources of weaning advice, comparisons between formal and informal advice, and associations with weaning timing in a survey of UK first-time mothers | Moore (2012) | Age – Cannot distinguish participants with infants under 24 months |
| An online survey of knowledge of the weaning guidelines, advice from health visitors and other factors that influence weaning timing in UK mothers | Moore (2014) | Methods - Qualitative data not presented only quantitative |
| Influence of weaning timing advice and associated weaning behaviours in a survey of black and minority ethnic groups in the UK | Moore (2014) | Methods - Qualitative data not presented only quantitative |
| Images of Childhood in Mothers' Accounts of Contemporary Childrearing | Murphy (2016) | Topic – Study did not mention complementary feeding |
| Parental concerns about complementary feeding: differences according to interviews with mothers with children of 7 and 13 months of age | Nielsen (2013) | Country – Study in Denmark |
| Study name | First author (date) | Reason for Exclusion |
| Infants' transition from milk to solid foods - the lived experiences of first-time parents | Norlyk (2019) | Country – Study in Denmark |
| Parents' Descriptions of Feeding Their Young Infants | Pados (2019) | Country – Study in USA and Canada |
| Bangladeshi women's experiences of infant feeding in the London Borough of Tower Hamlets | Rayment (2016) | Age – Cannot distinguish participants with infants under 24 months |
| A qualitative study of the infant feeding beliefs and behaviours of mothers with low educational attainment | Russell (2016) | Country – Study in Australia |
| Exchanging health advice in a virtual community: A story of tribalization | Rusu (2016) | Country – Study of online group of Romanian mothers |
| Infant feeding and the concept of early nutrition programming: a comparison of qualitative data from four European countries | Schmid (2009) | Participants - interviews with stakeholders not with parents or caregivers |
| Early weaning is related to weight and rate of weight gain in infancy | Sloan (2008) | Methods - Qualitative data not presented only quantitative |
| Mothers' experiences with complementary feeding: Conventional and baby-led approaches | Swanepoel (2020) | Country – Study in Australia |
| A Mixed Methods Study to Explore the Effects of Program Design Elements and Participant Characteristics on Parents' Engagement With an mHealth Program to Promote Healthy Infant Feeding: The Growing Healthy Program | Taki (2019) | Country – Study conducted in Australia |
| Consumer Engagement in Mobile Application (App) Interventions Focused on Supporting Infant Feeding Practices for Early Prevention of Childhood Obesity | Taki (2019) | Country – Study conducted in Australia |
| Co-parenting and feeding in early childhood: Reflections of parent dyads on how they manage the developmental stages of feeding over the first three years | Thullen (2016) | Country – Study in USA |
| Factors influencing first-time mothers' introduction of complementary foods: a qualitative exploration | Walsh (2015) | Country – Study conducted in Austrailia |
