# Supplementary material for: Parental engagement with complementary feeding information in the United Kingdom: A qualitative evidence synthesis
Source: Matern Child Nutr. 2023 Aug 8;19(4):e13553. doi: 10.1111/mcn.13553 (PMC10483955; doi:10.1111/mcn.13553)
Supplement: Supplementary file 2 — Supporting information. [file MCN-19-e13553-s002.docx]

Supplementary data 2 - Critical appraisal using the CASP checklist for qualitative research

|  | 1. Was there a clear statement of the aims of the research? | 2. Is a qualitative  methodology  appropriate? | 3. Was the research design appropriate to address the aims of the research? | 4. Was the recruitment strategy appropriate to the aims of the research? | 5. Was the data collected in a way that addressed the research issue? | 6. Has the relationship between researcher and participants been adequately considered? | 7. Have ethical issues been taken into consideration? | 8. Was the data analysis sufficiently rigorous? | 9. Is there a clear statement of findings? |
| --- | --- | --- | --- | --- | --- | --- | --- | --- | --- |
|  |  |  |  |  |  |  |  |  |  |
| Study ID |  |  |  |  |  |  |  |  |  |
| Andrews (2014) | 🗸 | 🗸 | 🗸 | 🗸 | 🗸 | X | 🗸 | 🗸 | 🗸 |
| Arden (2015) | 🗸 | 🗸 | 🗸 | 🗸 | 🗸 | X | 🗸 | 🗸 | 🗸 |
| Barlow (2010) | 🗸 | 🗸 | 🗸 | 🗸 | 🗸 | X | 🗸 | 🗸 | 🗸 |
| Brown (2011) | X | 🗸 | 🗸 | 🗸 | 🗸 | X | 🗸 | 🗸 | 🗸 |
| Carstairs  (2017) | 🗸 | 🗸 | 🗸 | 🗸 | 🗸 | 🗸 | 🗸 | 🗸 | 🗸 |
| Garcia (2019) | 🗸 | 🗸 | 🗸 | 🗸 | 🗸 | X | 🗸 | 🗸 | 🗸 |
| Hoddinott/McInnes (2012) | 🗸 | 🗸 | 🗸 | 🗸 | 🗸 | X | 🗸 | 🗸 | 🗸 |
| Lakhanpaul (2020) | 🗸 | 🗸 | 🗸 | 🗸 | 🗸 | 🗸 | 🗸 | 🗸 | 🗸 |
| Lovelace  (2015) | 🗸 | 🗸 | 🗸 | 🗸 | 🗸 | 🗸 | 🗸 | 🗸 | 🗸 |
| McNally  (2020) | 🗸 | 🗸 | 🗸 | 🗸 | 🗸 | X | 🗸 | 🗸 | 🗸 |
| Redsell (2010) | 🗸 | 🗸 | 🗸 | 🗸 | 🗸 | X | 🗸 | 🗸 | 🗸 |
| Synott  (2007) | 🗸 | 🗸 | 🗸 | Can’t tell from the paper | 🗸 | X | 🗸 | 🗸 | X |
| Tully/Spyreli (2019) | 🗸 | 🗸 | 🗸 | 🗸 | 🗸 | 🗸 | 🗸 | 🗸 | 🗸 |
| Zhang (2019) | 🗸 | 🗸 | 🗸 | 🗸 | 🗸 | 🗸 | 🗸 | 🗸 | 🗸 |
